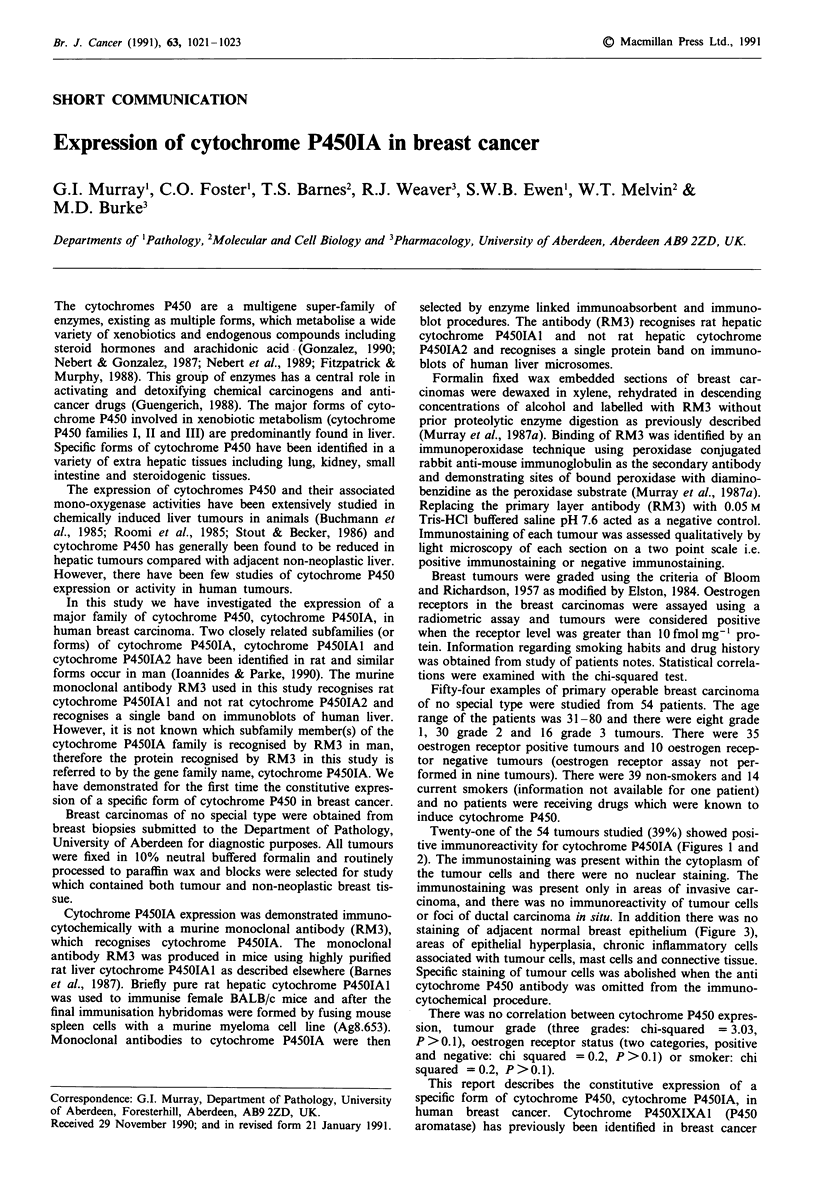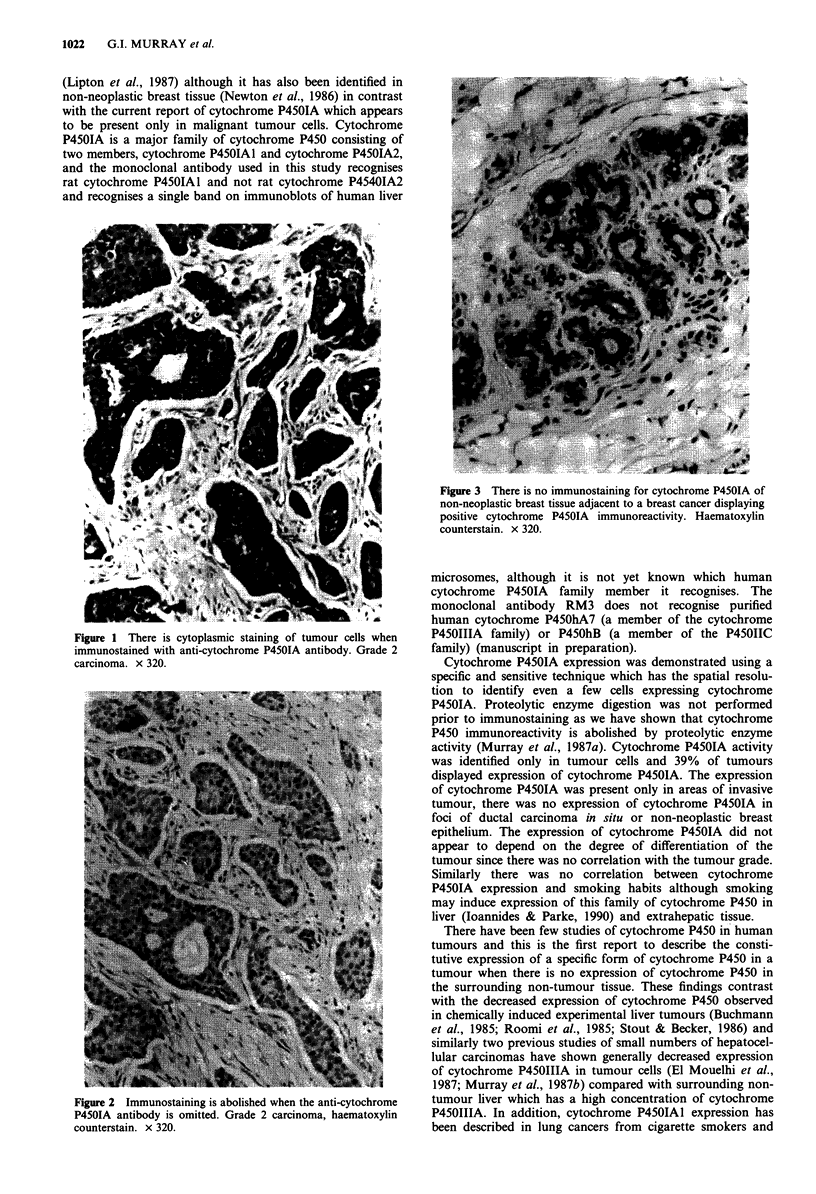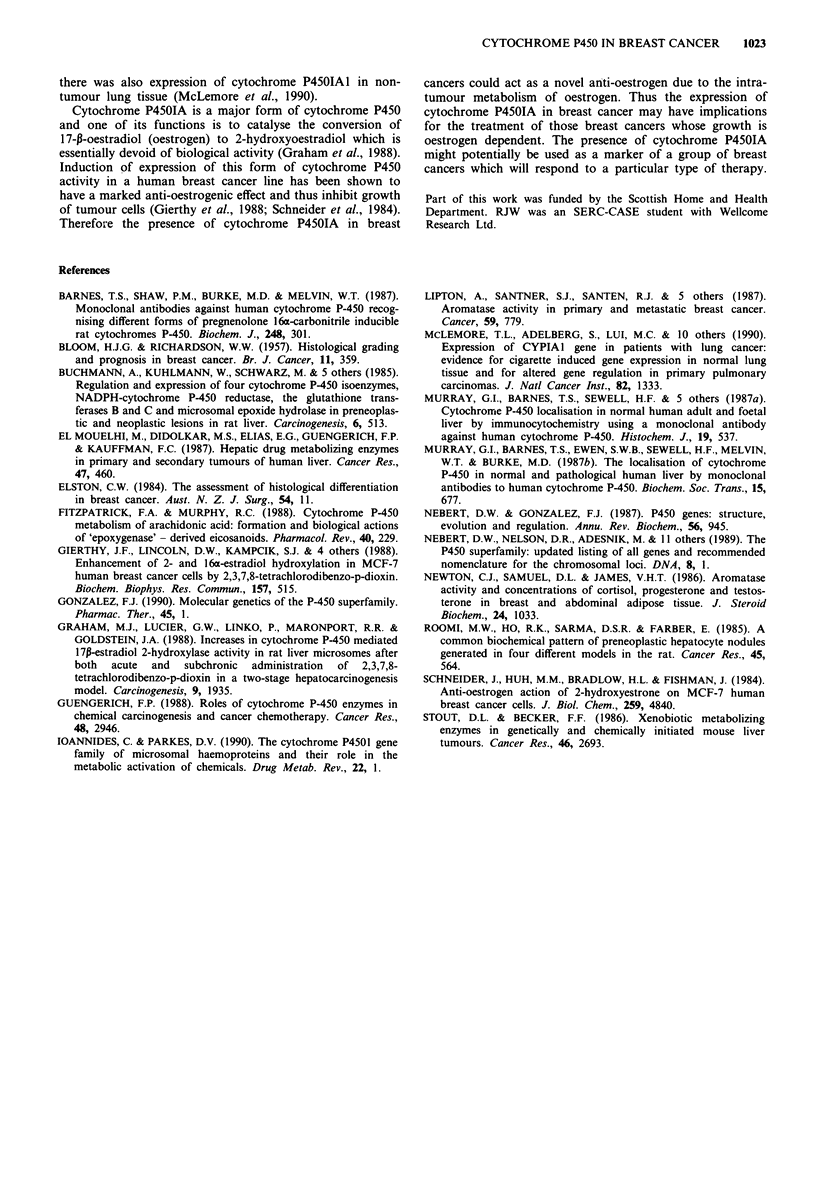# Expression of cytochrome P450IA in breast cancer.

**DOI:** 10.1038/bjc.1991.222

**Published:** 1991-06

**Authors:** G. I. Murray, C. O. Foster, T. S. Barnes, R. J. Weaver, S. W. Ewen, W. T. Melvin, M. D. Burke

**Affiliations:** Department of Pathology, University of Aberdeen, UK.

## Abstract

**Images:**


Br. J. Cancer (1991), 63, 1021–1023 © Macmillan Press Ltd., 1991

SHORT COMMUNICATION

### Expression of cytochrome P450IA in breast cancer

G.I. Murray[1], C.O. Foster[1], T.S. Barnes[2], R.J. Weaver[3], S.W.B. Ewen[1], W.T. Melvin[2] & M.D. Burke[3]

*Departments of [1]Pathology, [2]Molecular and Cell Biology and [3]Pharmacology, University of Aberdeen, Aberdeen AB9 2ZD, UK.*

The cytochromes P450 are a multigene super-family of enzymes, existing as multiple forms, which metabolise a wide variety of xenobiotics and endogenous compounds including steroid hormones and arachidonic acid (Gonzalez, 1990; Nebert & Gonzalez, 1987; Nebert *et al.*, 1989; Fitzpatrick & Murphy, 1988). This group of enzymes has a central role in activating and detoxifying chemical carcinogens and anticancer drugs (Guengerich, 1988). The major forms of cytochrome P450 involved in xenobiotic metabolism (cytochrome P450 families I, II and III) are predominantly found in liver. Specific forms of cytochrome P450 have been identified in a variety of extra hepatic tissues including lung, kidney, small intestine and steroidogenic tissues.

The expression of cytochromes P450 and their associated mono-oxygenase activities have been extensively studied in chemically induced liver tumours in animals (Buchmann *et al.*, 1985; Roomi *et al.*, 1985; Stout & Becker, 1986) and cytochrome P450 has generally been found to be reduced in hepatic tumours compared with adjacent non-neoplastic liver. However, there have been few studies of cytochrome P450 expression or activity in human tumours.

In this study we have investigated the expression of a major family of cytochrome P450, cytochrome P450IA, in human breast carcinoma. Two closely related subfamilies (or forms) of cytochrome P450IA, cytochrome P450IA1 and cytochrome P450IA2 have been identified in rat and similar forms occur in man (Ioannides & Parke, 1990). The murine monoclonal antibody RM3 used in this study recognises rat cytochrome P450IA1 and not rat cytochrome P450IA2 and recognises a single band on immunoblots of human liver. However, it is not known which subfamily member(s) of the cytochrome P450IA family is recognised by RM3 in man, therefore the protein recognised by RM3 in this study is referred to by the gene family name, cytochrome P450IA. We have demonstrated for the first time the constitutive expression of a specific form of cytochrome P450 in breast cancer.

Breast carcinomas of no special type were obtained from breast biopsies submitted to the Department of Pathology, University of Aberdeen for diagnostic purposes. All tumours were fixed in 10% neutral buffered formalin and routinely processed to paraffin wax and blocks were selected for study which contained both tumour and non-neoplastic breast tissue.

Cytochrome P450IA expression was demonstrated immunocytochemically with a murine monoclonal antibody (RM3), which recognises cytochrome P450IA. The monoclonal antibody RM3 was produced in mice using highly purified rat liver cytochrome P450IA1 as described elsewhere (Barnes *et al.*, 1987). Briefly pure rat hepatic cytochrome P450IA1 was used to immunise female BALB/c mice and after the final immunisation hybridomas were formed by fusing mouse spleen cells with a murine myeloma cell line (Ag8.653). Monoclonal antibodies to cytochrome P450IA were then selected by enzyme linked immunoabsorbent and immunoblot procedures. The antibody (RM3) recognises rat hepatic cytochrome P450IA1 and not rat hepatic cytochrome P450IA2 and recognises a single protein band on immunoblots of human liver microsomes.

Formalin fixed wax embedded sections of breast carcinomas were dewaxed in xylene, rehydrated in descending concentrations of alcohol and labelled with RM3 without prior proteolytic enzyme digestion as previously described (Murray *et al.*, 1987*a*). Binding of RM3 was identified by an immunoperoxidase technique using peroxidase conjugated rabbit anti-mouse immunoglobulin as the secondary antibody and demonstrating sites of bound peroxidase with diaminobenzidine as the peroxidase substrate (Murray *et al.*, 1987*a*). Replacing the primary layer antibody (RM3) with 0.05 M Tris-HCl buffered saline pH 7.6 acted as a negative control. Immunostaining of each tumour was assessed qualitatively by light microscopy of each section on a two point scale i.e. positive immunostaining or negative immunostaining.

Breast tumours were graded using the criteria of Bloom and Richardson, 1957 as modified by Elston, 1984. Oestrogen receptors in the breast carcinomas were assayed using a radiometric assay and tumours were considered positive when the receptor level was greater than 10 fmol $mg^{-1}$ protein. Information regarding smoking habits and drug history was obtained from study of patients notes. Statistical correlations were examined with the chi-squared test.

Fifty-four examples of primary operable breast carcinoma of no special type were studied from 54 patients. The age range of the patients was 31–80 and there were eight grade 1, 30 grade 2 and 16 grade 3 tumours. There were 35 oestrogen receptor positive tumours and 10 oestrogen receptor negative tumours (oestrogen receptor assay not performed in nine tumours). There were 39 non-smokers and 14 current smokers (information not available for one patient) and no patients were receiving drugs which were known to induce cytochrome P450.

Twenty-one of the 54 tumours studied (39%) showed positive immunoreactivity for cytochrome P450IA (Figures 1 and 2). The immunostaining was present within the cytoplasm of the tumour cells and there were no nuclear staining. The immunostaining was present only in areas of invasive carcinoma, and there was no immunoreactivity of tumour cells or foci of ductal carcinoma *in situ*. In addition there was no staining of adjacent normal breast epithelium (Figure 3), areas of epithelial hyperplasia, chronic inflammatory cells associated with tumour cells, mast cells and connective tissue. Specific staining of tumour cells was abolished when the anti cytochrome P450 antibody was omitted from the immunocytochemical procedure.

There was no correlation between cytochrome P450 expression, tumour grade (three grades: chi-squared = 3.03, $P > 0.1$), oestrogen receptor status (two categories, positive and negative: chi squared = 0.2, $P > 0.1$) or smoker: chi squared = 0.2, $P > 0.1$).

This report describes the constitutive expression of a specific form of cytochrome P450, cytochrome P450IA, in human breast cancer. Cytochrome P450XIXA1 (P450 aromatase) has previously been identified in breast cancer

Correspondence: G.I. Murray, Department of Pathology, University of Aberdeen, Foresterhill, Aberdeen, AB9 2ZD, UK.
Received 29 November 1990; and in revised form 21 January 1991.

(Lipton *et al.*, 1987) although it has also been identified in non-neoplastic breast tissue (Newton *et al.*, 1986) in contrast with the current report of cytochrome P450IA which appears to be present only in malignant tumour cells. Cytochrome P450IA is a major family of cytochrome P450 consisting of two members, cytochrome P450IA1 and cytochrome P450IA2, and the monoclonal antibody used in this study recognises rat cytochrome P450IA1 and not rat cytochrome P4540IA2 and recognises a single band on immunoblots of human liver

**Figure 1** There is cytoplasmic staining of tumour cells when immunostained with anti-cytochrome P450IA antibody. Grade 2 carcinoma. × 320.

**Figure 2** Immunostaining is abolished when the anti-cytochrome P450IA antibody is omitted. Grade 2 carcinoma, haematoxylin counterstain. × 320.

**Figure 3** There is no immunostaining for cytochrome P450IA of non-neoplastic breast tissue adjacent to a breast cancer displaying positive cytochrome P450IA immunoreactivity. Haematoxylin counterstain. × 320.

microsomes, although it is not yet known which human cytochrome P450IA family member it recognises. The monoclonal antibody RM3 does not recognise purified human cytochrome P450hA7 (a member of the cytochrome P450IIIA family) or P450hB (a member of the P450IIC family) (manuscript in preparation).

Cytochrome P450IA expression was demonstrated using a specific and sensitive technique which has the spatial resolution to identify even a few cells expressing cytochrome P450IA. Proteolytic enzyme digestion was not performed prior to immunostaining as we have shown that cytochrome P450 immunoreactivity is abolished by proteolytic enzyme activity (Murray *et al.*, 1987*a*). Cytochrome P450IA activity was identified only in tumour cells and 39% of tumours displayed expression of cytochrome P450IA. The expression of cytochrome P450IA was present only in areas of invasive tumour, there was no expression of cytochrome P450IA in foci of ductal carcinoma *in situ* or non-neoplastic breast epithelium. The expression of cytochrome P450IA did not appear to depend on the degree of differentiation of the tumour since there was no correlation with the tumour grade. Similarly there was no correlation between cytochrome P450IA expression and smoking habits although smoking may induce expression of this family of cytochrome P450 in liver (Ioannides & Parke, 1990) and extrahepatic tissue.

There have been few studies of cytochrome P450 in human tumours and this is the first report to describe the constitutive expression of a specific form of cytochrome P450 in a tumour when there is no expression of cytochrome P450 in the surrounding non-tumour tissue. These findings contrast with the decreased expression of cytochrome P450 observed in chemically induced experimental liver tumours (Buchmann *et al.*, 1985; Roomi *et al.*, 1985; Stout & Becker, 1986) and similarly two previous studies of small numbers of hepatocellular carcinomas have shown generally decreased expression of cytochrome P450IIIA in tumour cells (El Mouelhi *et al.*, 1987; Murray *et al.*, 1987*b*) compared with surrounding non-tumour liver which has a high concentration of cytochrome P450IIIA. In addition, cytochrome P450IA1 expression has been described in lung cancers from cigarette smokers and there was also expression of cytochrome P450IA1 in non-tumour lung tissue (McLemore *et al.*, 1990).

Cytochrome P450IA is a major form of cytochrome P450 and one of its functions is to catalyse the conversion of 17-β-oestradiol (oestrogen) to 2-hydroxyoestradiol which is essentially devoid of biological activity (Graham *et al.*, 1988). Induction of expression of this form of cytochrome P450 activity in a human breast cancer line has been shown to have a marked anti-oestrogenic effect and thus inhibit growth of tumour cells (Gierthy *et al.*, 1988; Schneider *et al.*, 1984). Therefore the presence of cytochrome P450IA in breast cancers could act as a novel anti-oestrogen due to the intra-tumour metabolism of oestrogen. Thus the expression of cytochrome P450IA in breast cancer may have implications for the treatment of those breast cancers whose growth is oestrogen dependent. The presence of cytochrome P450IA might potentially be used as a marker of a group of breast cancers which will respond to a particular type of therapy.

Part of this work was funded by the Scottish Home and Health Department. RJW was an SERC-CASE student with Wellcome Research Ltd.

### References

BARNES, T.S., SHAW, P.M., BURKE, M.D. & MELVIN, W.T. (1987). Monoclonal antibodies against human cytochrome P-450 recognising different forms of pregnenolone 16α-carbonitrile inducible rat cytochromes P-450. *Biochem. J.*, **248**, 301.

BLOOM, H.J.G. & RICHARDSON, W.W. (1957). Histological grading and prognosis in breast cancer. *Br. J. Cancer*, **11**, 359.

BUCHMANN, A., KUHLMANN, W., SCHWARZ, M. & 5 others (1985). Regulation and expression of four cytochrome P-450 isoenzymes, NADPH-cytochrome P-450 reductase, the glutathione transferases B and C and microsomal epoxide hydrolase in preneoplastic and neoplastic lesions in rat liver. *Carcinogenesis*, **6**, 513.

EL MOUELHI, M., DIDOLKAR, M.S., ELIAS, E.G., GUENGERICH, F.P. & KAUFFMAN, F.C. (1987). Hepatic drug metabolizing enzymes in primary and secondary tumours of human liver. *Cancer Res.*, **47**, 460.

ELSTON, C.W. (1984). The assessment of histological differentiation in breast cancer. *Aust. N. Z. J. Surg.*, **54**, 11.

FITZPATRICK, F.A. & MURPHY, R.C. (1988). Cytochrome P-450 metabolism of arachidonic acid: formation and biological actions of 'epoxygenase' – derived eicosanoids. *Pharmacol. Rev.*, **40**, 229.

GIERTHY, J.F., LINCOLN, D.W., KAMPCIK, S.J. & 4 others (1988). Enhancement of 2- and 16α-estradiol hydroxylation in MCF-7 human breast cancer cells by 2,3,7,8-tetrachlorodibenzo-p-dioxin. *Biochem. Biophys. Res. Commun.*, **157**, 515.

GONZALEZ, F.J. (1990). Molecular genetics of the P-450 superfamily. *Pharmac. Ther.*, **45**, 1.

GRAHAM, M.J., LUCIER, G.W., LINKO, P., MARONPORT, R.R. & GOLDSTEIN, J.A. (1988). Increases in cytochrome P-450 mediated 17β-estradiol 2-hydroxylase activity in rat liver microsomes after both acute and subchronic administration of 2,3,7,8-tetrachlorodibenzo-p-dioxin in a two-stage hepatocarcinogenesis model. *Carcinogenesis*, **9**, 1935.

GUENGERICH, F.P. (1988). Roles of cytochrome P-450 enzymes in chemical carcinogenesis and cancer chemotherapy. *Cancer Res.*, **48**, 2946.

IOANNIDES, C. & PARKES, D.V. (1990). The cytochrome P4501 gene family of microsomal haemoproteins and their role in the metabolic activation of chemicals. *Drug Metab. Rev.*, **22**, 1.

LIPTON, A., SANTNER, S.J., SANTEN, R.J. & 5 others (1987). Aromatase activity in primary and metastatic breast cancer. *Cancer*, **59**, 779.

MCLEMORE, T.L., ADELBERG, S., LUI, M.C. & 10 others (1990). Expression of CYPIA1 gene in patients with lung cancer: evidence for cigarette induced gene expression in normal lung tissue and for altered gene regulation in primary pulmonary carcinomas. *J. Natl Cancer Inst.*, **82**, 1333.

MURRAY, G.I., BARNES, T.S., SEWELL, H.F. & 5 others (1987*a*). Cytochrome P-450 localisation in normal human adult and foetal liver by immunocytochemistry using a monoclonal antibody against human cytochrome P-450. *Histochem. J.*, **19**, 537.

MURRAY, G.I., BARNES, T.S., EWEN, S.W.B., SEWELL, H.F., MELVIN, W.T. & BURKE, M.D. (1987*b*). The localisation of cytochrome P-450 in normal and pathological human liver by monoclonal antibodies to human cytochrome P-450. *Biochem. Soc. Trans.*, **15**, 677.

NEBERT, D.W. & GONZALEZ, F.J. (1987). P450 genes: structure, evolution and regulation. *Annu. Rev. Biochem.*, **56**, 945.

NEBERT, D.W., NELSON, D.R., ADESNIK, M. & 11 others (1989). The P450 superfamily: updated listing of all genes and recommended nomenclature for the chromosomal loci. *DNA*, **8**, 1.

NEWTON, C.J., SAMUEL, D.L. & JAMES, V.H.T. (1986). Aromatase activity and concentrations of cortisol, progesterone and testosterone in breast and abdominal adipose tissue. *J. Steroid Biochem.*, **24**, 1033.

ROOMI, M.W., HO, R.K., SARMA, D.S.R. & FARBER, E. (1985). A common biochemical pattern of preneoplastic hepatocyte nodules generated in four different models in the rat. *Cancer Res.*, **45**, 564.

SCHNEIDER, J., HUH, M.M., BRADLOW, H.L. & FISHMAN, J. (1984). Anti-oestrogen action of 2-hydroxyestrone on MCF-7 human breast cancer cells. *J. Biol. Chem.*, **259**, 4840.

STOUT, D.L. & BECKER, F.F. (1986). Xenobiotic metabolizing enzymes in genetically and chemically initiated mouse liver tumours. *Cancer Res.*, **46**, 2693.